# Evolutionary consequences of genomic deletions and insertions in the woolly mammoth genome

**DOI:** 10.1016/j.isci.2022.104826

**Published:** 2022-08-01

**Authors:** Tom van der Valk, Marianne Dehasque, J. Camilo Chacón-Duque, Nikolay Oskolkov, Sergey Vartanyan, Peter D. Heintzman, Patrícia Pečnerová, David Díez-del-Molino, Love Dalén

**Affiliations:** 1 Department of Bioinformatics and Genetics, Swedish Museum of Natural History, Stockholm, Sweden; 2 Centre for Palaeogenetics, Svante Arrhenius väg 20C, SE-10691 Stockholm, Sweden; 3 Department of Zoology, Stockholm University, SE-10691, Stockholm, Sweden; 4 Department of Archaeology and Classical Studies, Stockholm University, SE-1069, Stockholm, Sweden; 5 Department of Biology, National Bioinformatics Infrastructure Sweden, Science for Life Laboratory, Lund University, Sweden; 6 North-East Interdisciplinary Scientific Research Institute n.a. N.A. Shilo, Far East Branch, Russian Academy of Sciences (NEISRI FEB RAS), Magadan, Russia; 7 The Arctic University Museum of Norway, UiT - The Arctic University of Norway, Tromsø, Norway; 8 Section for Computational and RNA Biology, Department of Biology, University of Copenhagen, Copenhagen, Denmark

**Keywords:** Natural sciences, Biological sciences, Zoology, Evolutionary biology, Phylogenetics, Bioinformatics

## Abstract

Woolly mammoths had a set of adaptations that enabled them to thrive in the Arctic environment. Many mammoth-specific single nucleotide polymorphisms (SNPs) responsible for unique mammoth traits have been previously identified from ancient genomes. However, a multitude of other genetic variants likely contributed to woolly mammoth evolution. In this study, we sequenced two woolly mammoth genomes and combined these with previously sequenced mammoth and elephant genomes to conduct a survey of mammoth-specific deletions and indels. We find that deletions are highly enriched in non-coding regions, suggesting selection against structural variants that affect protein sequences. Nonetheless, at least 87 woolly mammoth genes contain deletions or indels that modify the coding sequence, including genes involved in skeletal morphology and hair growth. These results suggest that deletions and indels contributed to the unique phenotypic adaptations of the woolly mammoth, and were potentially critical to surviving in its natural environment.

## Highlights

• Two new high-quality woolly mammoth genomes have been generated
• A new method was used to identify deletions and insertions in woolly mammoths
• At least 87 genes have been affected by deletions or indels in the mammoth lineage
• Genes involved in skeletal morphology and hair growth are affected by deletions

## Graphical abstract

**Figure:**
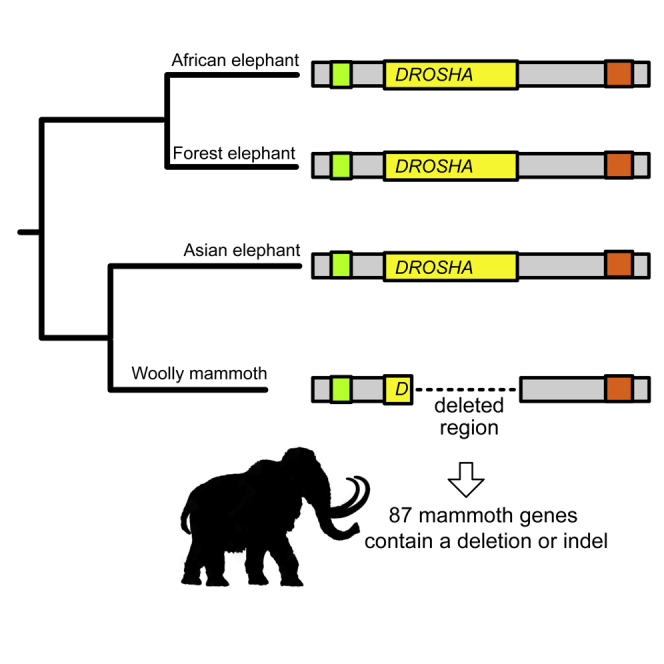

## Introduction

A key challenge in biology is to understand the genetic basis for the evolution of phenotypic adaptations. Ultimately, natural selection occurs at the level of DNA variation, and many processes are known to cause genetic variation within and among species. Among these variations are single nucleotide polymorphisms (SNPs), changes in methylation patterns, and structural variants, such as gene copy numbers and sequence insertions or deletions. In this study, we aimed to identify large deletions (above 500 bp) and smaller indels (below 25 bp) that might have contributed to the unique adaptations of the iconic woolly mammoth (*Mammuthus primigenius*). The woolly mammoth was the last surviving species of *Mammuthus*, a genus which originated in Africa ∼5–3 million years ago (Lister and Bahn, 2007; Palkopoulou et al., 2018). Whereas the living relatives of the woolly mammoth, the Asian and African elephants, are only found in tropical and subtropical environments, mammoths were adapted to the cold, dry steppe-tundra of the Northern Hemisphere high latitudes with average winter temperatures ranging from −30°C to −50°C (MacDonald et al., 2012). To cope with their extreme environment, woolly mammoths had a set of unique adaptations to minimize heat loss, such as thick fur, small ears and tails, as well as a thick layer of fat under the skin as energy fat reservoir during the winter (Fisher et al., 2012). This made the woolly mammoth exceptionally well adapted to the extreme Arctic environment, resulting in a circumpolar distribution, and becoming a keystone component of steppe-tundra ecosystems (Gill, 2014). High-quality genomes of the woolly mammoth were first sequenced in 2015 (Palkopoulou et al., 2015). Since then, multiple studies have aimed to characterize the unique adaptations of this species at the molecular level (Lynch et al., 2015; van der Valk et al., 2021; Smith et al., 2017). These studies identified a comprehensive set of protein-altering SNPs in the woolly mammoth genomes that are not present in either African or Asian elephant genomes, thus likely coding for some of the unique woolly mammoth traits.

Although SNPs are a major driver of genetic adaptations (1000 Genomes Project Consortium, 2015), they likely convey a subset of all molecular changes that made the woolly mammoth able to thrive in the Arctic. Structural genetic variants, such as deletions and insertions (indels), are known to have a role in the adaptation and speciation of vertebrates (Mérot et al., 2020). In this study, we aimed to identify genomic indels unique for woolly mammoths, with a focus on those causing inactivation (loss) or alteration of the ancestral protein sequence. Partially hampered by the absence of enough high-quality mammoth genomes and the difficulty of identifying structural variation from ancient DNA molecules, which are highly fragmented, systematic analyses of molecular changes other than SNPs have thus far been challenging to conduct. In order to more confidently call indels and deletions fixed in the woolly mammoth lineage, we sequenced two new Siberian woolly mammoth specimens to 13.8X and 13.9X coverage, and combined these with three previously published genomes sequenced to high coverage (11X–22X) (see methods, Table S1). These five woolly mammoths lived at varying times, ranging from 44,200 to 4,300 years ago, and represent different woolly mammoth genetic clades (Table S1). The diversity in genetic ancestry and age of these genomes ensures that the majority of deletions and indels fixed among them likely represent variants that were fixed or at high frequency across all woolly mammoths from the Late Pleistocene (126,000 to 11,700 years ago). To identify indels unique to the mammoth lineage, we included six previously published genomes of the closest living relatives of the woolly mammoths, the Asian elephant (*elephas maximus*), as well as 24 African savanna (*Loxodonta africana*) and forest elephant (*Loxodonta cyclotis*) genomes as comparative outgroups (Table S1 and Figure S1).

## Results

Due to the fragmented nature of ancient DNA, the majority of mammoth sequence reads are below 100 base pairs (bp; Table S1). As shorter reads are prone to being misaligned, we used a conservative filter that only included sequence reads of at least 50 bp in length and mapped these to the African savanna elephant reference genome (LoxAfr4; see methods), the closest woolly mammoth relative for which a high-quality reference is available. Next, we identified all reference genome regions where sequence reads of at least 50 bp can be confidently mapped, leaving 82% of the genome (2.3 billion bases) available for further downstream analyses (see methods , Figure S2). Short indels (below 25 bp) were then identified using GATK4, which uses a local de-novo assembly approach, improving the accuracy of indel identification over traditional variant callers (Poplin et al., 2018). Second, we identified deletions larger than 500 bp by employing an overlapping window-based search of regions without sequence read support (e.g. regions without read coverage) among the five woolly mammoth genomes but with good sequence read support among all African and Asian elephant genomes (see methods).

We identified 89,571 mammoth-specific short deletions (less than 25 bp), encompassing a total of 307,353 deleted bases (Figure S3). Although insertions are more challenging to detect, due to the nature of short-read sequencing technologies, we nonetheless identified 44,078 mammoth-specific short insertions comprising a total of 80,869 bp (Figure S3). Although some of these deletions and insertions could be the result of a structural variant mutation in the outgroup elephant genomes rather than in the mammoth lineage, such structural variants must have evolved independently in both the African and Asian elephant lineages and would thus be exceedingly rare. Additionally, using our window-based coverage approach, we identified 749 deletions longer than 500 bp, corresponding to a total of 2.9 million deleted bases (Table S2 and Figure S4). Thus, since the genetic divergence of the *Mammuthus* and *Elephas* lineages, between 5.2 and 2.2 million years ago (Palkopoulou et al., 2018), indels and deletions have shaped around 0.1% of the mammoth genome. Since we employed highly conservative methods for short indel and large deletion identification, this number likely represents a lower boundary. Although we used a reasonably large set of mammoth and elephant genomes to identify indels fixed among mammoths, the inclusion of additional mammoth genomes in future studies might reveal that some of the identified indels were at high frequency but not fixed throughout the entire woolly mammoth population. By randomly subsampling the number of mammoth genomes included in our analysis and comparing the total number of identified fixed indels as a function of sample size, we estimate that more than 90% of indels fixed among our five genomes were most likely also fixed among all other Late Pleistocene woolly mammoths (see methods, figure S5).

**Figure 1:**
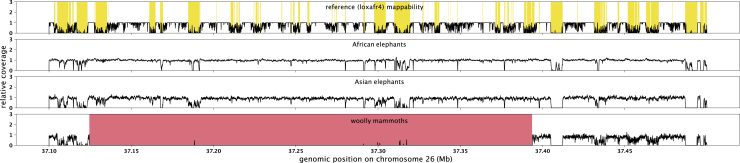
A woolly mammoth-specific 269 kb deletion on chromosome 26 Y axis shows the relative coverage across the depicted region, averaged across all genomes for each of the species. Whereas African and Asian elephants all have expected read depth in the red region, this region is devoid of reads among the woolly mammoth genomes. A few small coverage peaks in the woolly mammoth track can be seen, but these are in regions of low mappability (yellow boxes in the top track) and thus most likely represent misalignments.

**Figure 2:**
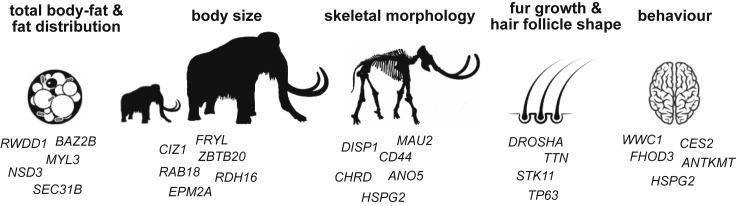
Mammalian phenotype associations for the genes with coding sequence changes in woolly mammoths as a result of indels and large deletions Phenotype associations were obtained from studies using mouse models. Disruption of the genes results in most cases in multiple different phenotypic effects (Table S6). We grouped genes by their phenotypic effects most relevant to mammoth adaptation. Since the function of these genes might not translate one-to-one into mammoths, these groupings should be interpreted with caution.

**Figure 3:**
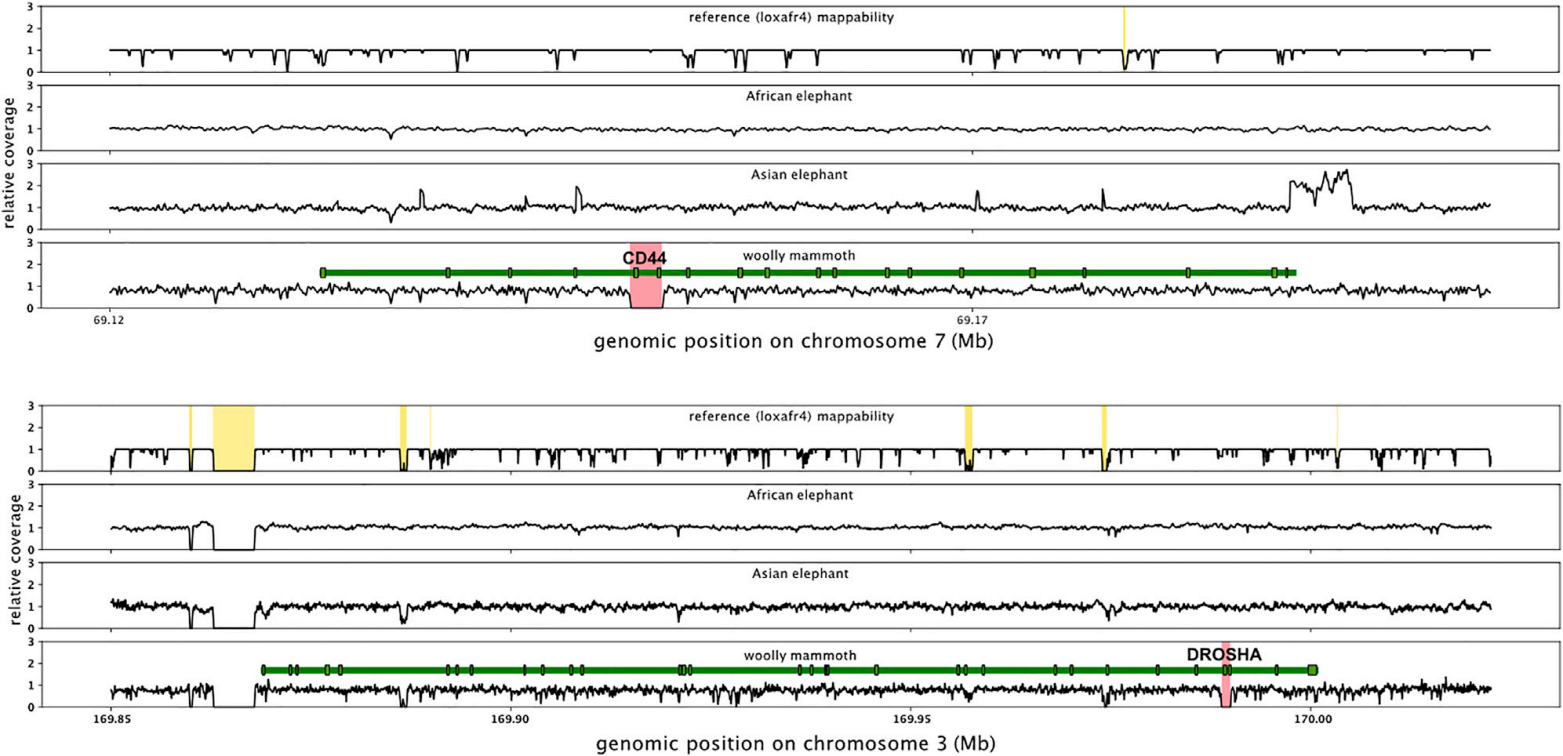
Woolly mammoth-specific deletions affecting exons of the CD44 and DROSHA genes Y axis shows the relative coverage across the depicted region, averaged across all genomes for each of the species. The dark green bars depict the gene position from start to end and light green boxes with gray outlines depict exons. In yellow shades are regions of low reference mappability and red shades depict mammoth-specific deletions.

Of the deletions present only in the woolly mammoths and in none of the other elephant genomes, 41 deletions are larger than 10 kb, with the largest deletion on chromosome 26 comprising a 269 kb region devoid of genes (Figures 1 and S4). Such large deletions are not uncommon for mammals and have also been described in, for example, great apes (Kronenberg et al., 2018). We find that indels are significantly enriched within gene-devoid regions, being 1.9 times more common in intergenic regions than in introns than would be expected under a random distribution (fisher’s exact test p < 0.001) and 9.1 times more common in intergenic regions than in exons (fisher’s exact test p < 0.001) (Table S3). This suggests that throughout mammoth evolution there was selection against indels that affect coding sequences. Nonetheless, we identified 87 genes for which at least one of the exons was affected by either a large deletion or a frameshift/stop-start loss-causing indel, and thus likely impacted gene function (Tables S4 and S5). Gene losses as a consequence of indels and deletions can be adaptive (Helsen et al., 2020) and multiple case studies investigating the fate of such variants have uncovered associations between gene loss and mammalian phenotypes under positive selection (Brawand et al., 2008; Meredith et al., 2014; Fang et al., 2014). In laboratory selection experiments, gene loss is a frequent cause of adaptations to various environmental conditions (Hottes et al., 2013). Given that we focused on those indels and large deletions that are fixed among woolly mammoths, the majority of these protein-altering variants likely conveyed adaptive effects and may have been under positive selection at some point during mammoth evolution. We did not find specific biological functions overrepresented among these genes (see methods), but many of the affected genes are related to known mammoth-specific phenotypes, such as total body-fat and fat distribution (*EPM2A*, *RDH16*, and *SEC31B*), fur growth and hair follicle shape and size (*CD34*, *DROSHA*, and *TP63*), skeletal morphology (*CD44*, *ANO5*, and *HSPG2*), ear morphology (*ILDR1* and *CHRD*), and body temperature (*CES2*) (Figure 2). In addition, we find several genes associated with body size (*ZBTB20*, *CIZ1*, and *TTN*), which might have been involved in the decreasing size of woolly mammoths during the late Pleistocene (Lister and Bahn, 2007). Among the genes for which a significant part of exome sequences are deleted are *CD44* and *DROSHA* (Figure 3). *CD44*, previously shown to be mutated in woolly mammoths (Smith et al., 2017), has a deletion overlapping the complete 5th and 6th exon (Figure 3). The *CD44* gene encodes a cell surface glycoprotein and the majority of the amino acid sequence is highly conserved between mammalian species (Goodison et al., 1999). In mouse models, *CD44* mediates the diameter and length of the tibia bone (Goodison et al., 1999). Mammoth limb bones are known to be stouter than the limb bones of modern day elephants with a reduced hind-to-fore leg ratio in mammoths compared to elephants and the deletion in the CD44 gene might play a part in this difference (Haynes, 1993). The *DROSHA gene*, which has its 29th and part of its 30th exon deleted (Figure 3), encodes a ribonuclease enzyme that executes the initiation step of micro-RNA processing in the cell nucleus. In mouse models, *DROSHA* is involved in hair follicle growth and shape and might thus have a role in fur growth in woolly mammoths (Teta et al., 2012).

Among genes containing frameshift-causing indels, we found *RDH16* and *ANO5*. The *RDH16* gene, which encodes a 317 amino-acid long retinol dehydrogenase, has a frameshift causing deletion at the 75th amino acid position. *RDH16* inactivation produces mice with increased vitamin A storage in the liver and kidney. Some high-latitude animals, such as polar bear, moose, arctic fox, and bearded seal, demonstrate no signs of vitamin A toxicity despite having 10–20 times the level of vitamin A in their livers as other animals (Russell, 1967; Senoo et al., 2012; Rodahl and Moore, 1943). This ability to efficiently store higher amounts of vitamin A may contribute to their survival during the extreme winter environment of the Arctic. *RDH16-*null mice also grow longer than the wild type, and have increased weight and adiposity, when restricted in vitamin A. Liver, kidneys, and multiple fat pads also increase in weight in *RDH16*-null mice (Zhang et al., 2007). We speculate that the frameshift in the RDH16 gene thus played a role in vitamin A storage and the associated phenotypic changes in woolly mammoths. The *ANO5* gene encodes for the 904 amino-acid long anoctamin-5 protein and contains a frameshift causing deletion at the 385th amino acid in mammoths. Homozygous *ANO5* knockout mice have some typical traits of humans with gnathodiaphyseal dysplasia syndrome, which is caused by mutations in *ANO5* (Marconi et al., 2013), including large jawbones, causing craniofacial changes, bowing tibia, and thickening of the femur and tibia. Consistent with these phenotypes, woolly mammoths are known to have massive jaws with taller skulls that are shorter from front to back to minimize the weight of the head and this might thus be partially mediated by the frameshift mutation in the ANO5 gene (Lister et al., 2005). All the inferred gene functions discussed here are based on mouse models, and thus do not necessarily translate accurately to their function in woolly mammoths. Nonetheless, the majority of genes are conserved in function across mammals, and it is thus likely that most of the deletion containing genes had comparable phenotypic functions in the mammoth lineage (Bradley et al., 2012).

## Discussion

It has previously been shown that genomic deletions had a role in the accumulation of genomic load in the last living mammoths, resulting in several dysfunctional genes in one of the last living woolly mammoth individuals, possibly aiding in their extinction (Rogers and Slatkin, 2017). Here, we show that indels and large deletions likely also contributed to adaptive phenotypic evolution in the woolly mammoths. Other forms of genomic variation, such as methylation patterns, chromosomal 3D structure, histone modifications, and gene expression changing variants remain to be discovered. The combination of all these variant types is ultimately the source of the unique adaptations that made woolly mammoths thrive in the Arctic environment. Several genetic engineering projects aimed at de-extinction of the woolly mammoth are currently ongoing (Shapiro, 2017). These are based on editing the genomes of living cells from Asian elephants, using for example CRISPR-Cas9 technology (Shapiro, 2017). Our results demonstrate that, should genome engineering be applied to extant elephants in order to recreate a woolly mammoth, a remarkable number of deletion and indel variants would have to be included in the editing process. We show that several genes with potentially important functions have been affected by these variants and it is likely that these genes would have consequences for the resulting biology of a resurrected “woolly mammoth”. Functional genetic pathways involved in fat distributions, fur growth, skeletal morphology, and behavior, are potentially critical to surviving in its natural or natural-like environment. Therefore, not including such genome edits could potentially preclude reintroduction and survival of a resurrected woolly mammoth in its natural habitat.

### Limitations of the study

Assessing the exact phenotypic impacts of DNA coding-sequence changes is a notoriously challenging task. Although we identified a set of genes containing deletions within their coding sequence, we can only speculate about their direct impact on woolly mammoth adaptations by using phenotypic data obtained from other mammalian, primary mouse model, studies. This comes with two main limitations, first the deletions in woolly mammoth coding sequences are not identical to the genetic variants observed in the mouse model studies, and it thus remains unclear if these variants impact the gene function in a similar way. Second, the function of a gene can be different between mouse and woolly mammoths. Introducing the mammoth genetic variants in either mouse models and/or elephant cell lines could thus improve the accuracy of the phenotypic inferences made in this study.

## STAR★Methods

### Key resources table

**Table:** 

REAGENT or RESOURCE	SOURCE	IDENTIFIER
**Biological samples**		

Tusk of the Woolly mammoth (*Mammuthus primigenius*)	Siberia	L163
Tusk of the Woolly mammoth (*Mammuthus primigenius*)	Siberia	M6

**Deposited data**		

Woolly mammoth (*Mammuthus primigenius*) resequencing datasets #1	This study; ENA (https://www.ebi.ac.uk/)	PRJEB52742
Woolly mammoth (*Mammuthus primigenius*) resequencing datasets #2	ENA (https://www.ebi.ac.uk/)	PRJEB7929
Woolly mammoth (*Mammuthus primigenius*) resequencing dataset #3	ENA (https://www.ebi.ac.uk/)	PRJDB4697
Reference genome of African elephant (*Loxodonta africana*)	Palkopoulou et al., (2018)	ftp://ftp.broadinstitute.org/pub/assemblies/mammals/elephant/loxAfr4/
Asian elephant (*Elephas maximus*) resequencing datasets	ENA (https://www.ebi.ac.uk/)	PRJEB24361
African elephant (*Loxodonta africana*) resequencing datasets #1	ENA (https://www.ebi.ac.uk/)	PRJEB24361
African elephant (*Loxodonta africana*) resequencing datasets #2	ENA (https://www.ebi.ac.uk/)	PRJNA622303
African elephant (*Loxodonta africana*) resequencing datasets #3	ENA (https://www.ebi.ac.uk/)	PRJNA761769

**Software and algorithms**		

seqprep v1.2	John (2011)	https://github.com/jstjohn/SeqPrep
bwa v0.7.17	Li and Durbin, 2009	http://bio-bwa.sourceforge.net/
GATK v4.2.0.0	McKenna et al. (2010)	https://gatk.broadinstitute.org/hc/en-us
Samremovedup	Skoglund, 2020	github.com/pontussk/samremovedup
Trimmomatic v.0.32	Bolger et al. (2014)	http://www.usadellab.org/cms/?page=trimmomatic
picard MarkDuplicates v2.26.6	broadinstitute.github.io/picard/	broadinstitute.github.io/picard/
BEDTools v2.29.2	Quinlan and Hall (2010)	https://bedtools.readthedocs.io/en/latest/
samtools v1.14	Li et al., (2009)	http://www.htslib.org/
SciPy.v1.8.1	Virtanen et al., 2020	https://scipy.org/
GOrilla	Eden et al., (2009)	http://cbl-gorilla.cs.technion.ac.il/
aDNA-deletions	This study	https://github.com/tvandervalk/aDNA-deletions

### Resource availability

#### Lead contact

Further information and requests for resources and reagents should be directed to and will be fulfilled by the Lead Contact, Tom van der Valk (tom.vandervalk@nrm.se).

#### Materials availability

This study did not generate new unique reagents.

### Method details

#### Sample description, DNA extraction and sequencing of mammoth samples

The two new woolly mammoth samples presented in this study represent tooth fragments of woolly mammoths collected in Chukotka (L163) and on Wrangel Island (M6). The samples had been radiocarbon dated previously (Vartanyan et al., 2008; Palkopoulou et al., 2013) and had mean ages of 31.9 and 24.0 thousand calibrated years before present for the Chukotka and Wrangel Island samples, respectively. We performed DNA extractions in the clean ancient DNA laboratory at the Swedish Museum of Natural History, following standard aDNA procedures including wearing protective suits, face masks and gloves, regular cleaning of surfaces with sodium hypochlorite, UV-sterilization of tools, and including negative controls at all steps of the laboratory work. We used a hand-held low-speed Dremel drill to obtain approximately 50 mg of tooth powder, and we extracted DNA from the powder following a silica-based method (Ersmark et al., 2015). Dual indexed Illumina libraries were built for all extracts following a protocol for double-stranded DNA (Meyer and Kircher, 2010), including a uracil-DNA-glycosylase treatment (USER) step to remove deaminated cytosines. Indexing PCRs were prepared in independent indexing reactions per sample in order to minimise clonality, using 10 and 12 cycles for samples M6 and L163, respectively. Indexed libraries were cleaned to remove unligated adapters, adapter dimers, and long contaminating sequences by using size selection with Agencourt AMPure XP beads. Concentrations of cleaned libraries were estimated using a high-sensitivity DNA chip on the Bioanalyzer 2100 (Agilent). Indexed libraries were pooled together for each sample in equimolar concentrations. Each sample was sequenced on two lanes on an Illumina HiSeqX platform using paired end settings (2x150 bp).

#### Data processing

We downloaded the raw sequencing data from 33 previously published high-coverage elephantid genomes, which includes three woolly mammoths, six Asian elephants, 22 African savanna elephants, and two African forest elephants (Table S1) (Palkopoulou et al., 2015, 2018; Campbell-Staton et al., 2021; Tollis et al., 2021; Yamagata et al., 2019), and processed them together with the raw sequencing data from the two newly sequenced woolly mammoth samples. We used the GenErode pipeline (Kutschera et al., 2022) with minor modifications to map both ancient and modern data to a composite reference consisting of three concatenated genomes: the nuclear genome of the African savannah elephant (LoxAfr4, Broad Institute), the woolly mammoth mitogenome (Krause et al., 2006), and the human reference genome (hg19). Including the human reference as a decoy during mapping ensures that human contaminant reads are effectively filtered out before downstream analysis (Feuerborn et al., 2020) including the woolly mammoth mitochondria in the reference allowed us to avoid conserved regions between the nuclear and the mitochondrial genome (numts) from biassing the coverage profiles. Briefly, for the ancient samples, we trimmed Illumina adapters from the raw data and merged paired-end reads, as recommended for ancient DNA (Kircher, 2012), using *Seqprep* v1.2 (John, 2011) with default settings (a minimal overlap of 15 bp between the paired-end reads and excluding merged reads that are shorter than 30 bp). Following (Palkopoulou et al., 2015), we used a minor modification in *seqprep’s* source code that chooses the best quality score of bases in the merged region instead of aggregating them. We then mapped the merged reads to a concatenated reference using *bwa aln* v0.7.17 (Li, 2013) with the seed deactivated (-l 16,500), allowing for more substitutions (-n 0.01) and up to two gaps (-o 2) as recommend for mapping ancient DNA reads (Schubert et al., 2012). Next, we merged realigned mapped reads around indels using *GATK IndelRealigner* v3.4.0 (McKenna et al., 2010) and removed PCR duplicates from the alignments using a custom Python script (github.com/pontussk/samremovedup) that identifies reads as duplicates only if they share identical start and end coordinates (Palkopoulou et al., 2015). For the modern samples, sequencing adapters were trimmed with *Trimmomatic v*.*0*.*32* (Bolger et al., 2014) using default settings. We then mapped the trimmed reads to the reference genome using BWA mem v.0.7.13 (Li, 2013), marked potential duplicates using *picard MarkDuplicates* v2.26.6 (broadinstitute.github.io/picard/), and realigned reads around indels as above. Finally, as short sequence reads have a higher chance of misalignment (Poullet and Orlando, 2020) and can thereby distort the coverage signal used to detect deletions, we conservatively filtered reads below the length of 50 bp in all downstream analysis. This resulted in a final average coverage for the woolly mammoth genomes of 11.8X (8.9–17.9X).

#### Mappability mask

Due to the presence of repetitive or highly similar regions in the African savanna elephant reference genome, short-length reads can sometimes map to two or more regions of the genome, and are thus filtered out from the alignments (see section 2.2). This typically results in a fraction of the genome without coverage, resulting in the same signal as obtained from true genomic deletions. To prevent identifying these “unmappable” regions as deletions we created a mappability mask. First, we fragmented the LoxAfr4 reference genome into overlapping, 50 bp reads, by sliding across the reference sequence in overlapping windows of 50 bp using a step size of 1 bp and recording the sequence for each window (https://github.com/tvandervalk/aDNA-deletions). We then mapped these reads back to the reference using the same parameters as used for mapping the ancient samples (see section 2.2) and filtered out all non-uniquely mapping reads. Next, we used a strict coverage threshold, recording all sites with depth below 25 as unmappable into a bed-file (Figure S2). All unmappable regions within 250 base pairs from each other were then merged into one larger region using BEDTools v2.29.2 merge (-d 250) (Quinlan and Hall, 2010). We restricted our analysis to the 27 autosomal chromosomes due to the low mappability of the unplaced contigs and sex chromosome.

#### Quantification and statistical analysis Indels

We used *GATK v4*.*2*.*0*.*0 HaplotypeCaller* to jointly call short (<25 bp) insertions and deletions (indels) across all elephant genomes. The GATK caller builds local de-novo assemblies of haplotypes and whenever a region showing signs of variation is encountered, existing mapping information is discarded and the reads in the region are de-novo reassembled. This increases the accuracy of variant calling, especially in regions that are traditionally difficult to assess, such as those regions containing indels (Hwang et al., 2015). Next we filtered out all low quality indels following the GATK-best-practice recommendations (*GATK VariantFiltration -filter "QD < 2*.*0" "QUAL < 30*.*0" “FS > 200*.*0" "ReadPosRankSum < -20*.*0"*). In addition, we filtered indels outside mappable regions (see section 2.3), those supported by less than five reads, and those sites with more than one variant among the mammoth genomes. Finally, we focussed on indels that were uniquely present and fixed in woolly mammoths by keeping only indels for which all African and Asian elephant genomes were homozygous for the reference allele and all mammoth genomes homozygous for the alternative allele.

#### Large deletions

In order to detect larger deletions (>500 bp) we made use of the genome coverage information across all samples. The underlying reasoning of our method is that genomic regions identified as mappable (see section 2.3) and not containing aligned reads among all five woolly mammoth genomes but normal read coverage in all African and Asian elephant genomes most likely represent mammoth-specific genomic deletions. To identify such regions, we first estimated the per site depth for each genome using *samtools v1*.*14 depth* (Li et al., 2009), filtering reads below mapping quality of 30 (-Q 30) and length 50 (-l 50). Next, we summed the total depth per site by species (e.g. summing up the total depth at a site for all five mammoth genomes). Whereas summing the depth of several samples from the same species does not affect the coverage of fixed deletions (which remains at zero), it increases the depth of the surrounding regions by the factor of genomes (assuming equal depth among the genomes). This results in increased power for distinguishing deletions from surrounding regions with average coverage (Figure 1). Summing the depth by species, excluding unmappable regions, resulted in an average total coverage per site of 633X, 173X and 59X for the merged African elephant, Asian elephant, and woolly mammoth datasets, respectively (Figure S1). Next, we employed an overlapping window-based search algorithm to identify regions without sequence read support (e.g. regions without read coverage) among the five woolly mammoth genomes but with good coverage support among the African and Asian elephant genomes. We merged all identified windows adjacent to each other using bedtools merge and additionally merged deletions into a single larger deletion if they were broken apart by an unmappable region (see section 2.3). In rare occasions, a deleted region contains aligned reads either due to misalignments or the mismapping of non-endogenous DNA sequences (see Figure 1). To account for such effects, we additionally merged large deletions into a single larger deletion if they were within 250 bp from each other and the region separating the two deletions had a coverage lower than 10% of the expected woolly mammoth coverage (e.g. <5.9X).

#### Distribution of deletions

We tested if deletions are less often found in coding regions than expected under a random distribution. First, we randomly sampled a base position 1 million times and counted how often these overlapped with either an exon, intron or intergenic region. We repeated this procedure, randomly sampling a base position within an indel 1 million times . Next, Fisher’s exact test was run on the obtained contingency tables using the SciPy.v1.8.1 python package.

#### Sample size effects on fixed indels

In this study, we used five woolly mammoth genomes and 30 elephant genomes to identify mammoth specific indels fixed among woolly mammoths. Even though our mammoth genomes come from different geographical locations and time points (Dehasque et al., 2021), a subset of the identified fixed indels might have been variable among other unsampled mammoth genomes. To estimate the fraction of indels that are likely fixed in our dataset due to sampling stochasticity, we used a subsampling approach in which we identified the total number of fixed, woolly mammoth specific indels when sampling one, two, three, four, or all five woolly mammoth genomes. For each number of genomes (e.g. 1 to 5), we subsampled all possible combinations of individuals to include the effects of genomes representing different mitochondrial clades and sample ages. Next, we estimated the exponential trendline using *R* and forecasted the number of fixed deletions we expect to find when including additional woolly mammoth genomes (Figure S5).

#### Indels and deletions affecting coding sequences

To predict the functional impact of the identified indels and large deletions, we lifted over the ensemble release-105 African elephant genome (LoxAfr3) annotation to our reference genome (LoxAfr4) using the UCSC liftover tool (Hinrichs et al., 2006). We then applied *vep* (variant effect predictor) to identify all indels affecting coding sequences (McLaren et al., 2016). For large deletions, we used BEDtools intersect to identify all genes where (part of) exons were deleted. We then conducted a gene ontology enrichment on all genes for which the woolly mammoth genomes carried one or more fixed derived indels or large deletions, using GOrilla (Eden et al., 2009). Finally, we obtained gene ontologies for all genes causing amino acid deletions and/or frameshifts in the protein-sequence using the Mouse Genome Informatics database (informatics.jax.org).

## Data Availability

Raw sequencing reads from whole genome sequencing of the two woolly mammoth samples have been deposited at the European Nucleotide Archive (ENA PRJEB52742). All code used for identifying deletions is available at: https://github.com/tvandervalk/aDNA-deletions.

## Supplemental information

Document S1. Figures S1–S6

Table S1. Genome statistics depicting general statistics such as coverage, endogenous content and average fragment length of all genomes used in this study, related to STAR methods

Table S2. Genomic coordinates of the identified large deletions fixed in all woolly mammoth genomes and absent in all other elephant genomes, related to figure 1

Table S3. General statistics on the identified indels fixed in all woolly mammoth genomes and absent in all other elephant genomes, related to figure 1

Table S4. A list of genes for which (part of) an exon is deleted in all woolly mammoth genomes, related to figure 1 and 3

Table S5. A list of genes containing protein coding changing indels fixed in all woolly mammoth genomes. Related to figure 2

Table S6. Gene-ontology terms for the genes for which (part of) an exon is deleted in all woolly mammoth genomes, related to figure 2
