# Supplementary material for: Evolutionary consequences of genomic deletions and insertions in the woolly mammoth genome
Source: iScience. 2022 Aug 1;25(8):104826. doi: 10.1016/j.isci.2022.104826 (PMC9382235; doi:10.1016/j.isci.2022.104826)
Supplement: Document S1. Figures S1–S6 [file mmc1.pdf]

iScience, Volume *25*

Supplemental information

Evolutionary consequences of genomic deletions

and insertions in the woolly mammoth genome

Tom van der Valk, Marianne Dehasque, J. Camilo Chacón-Duque, Nikolay Oskolkov, Sergey Vartanyan, Peter D. Heintzman, Patrícia Pečnerová, David Díez-del-Molino, and Love Dalén

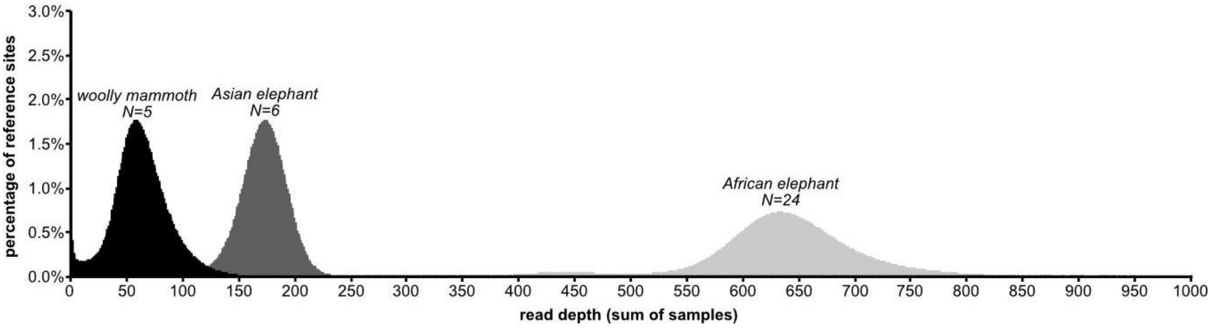

Figure S1. Read depth for the genomic samples used in this study. *Depth is shown as the sum of all reads that cover a site, separated per species (African savanna and African forest elephant were merged together due to the small sample size (N=2) of African forest elephants). Only reads with a minimum mapping quality of 30 and minimum length of 50 base pairs are included.*

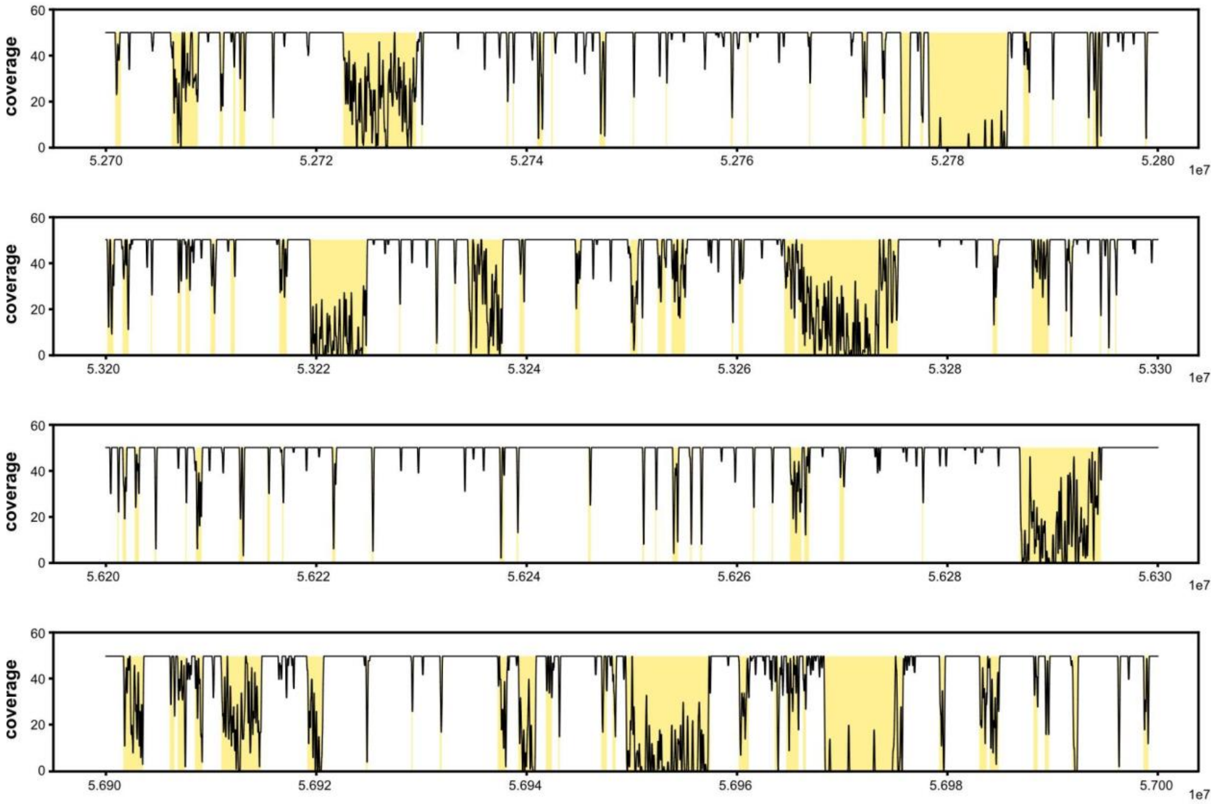

Figure S2. Read mappability of the African elephant reference genome (loxafr4). *Using a simulated dataset of sequence reads 50 bp in length mapped back to the reference we estimated genome mappability. An example region along chromosome 21 is shown, with in yellow those regions filtered out during all our analysis due to poor mappability and thus being unreliable for deletions and indel calling.*

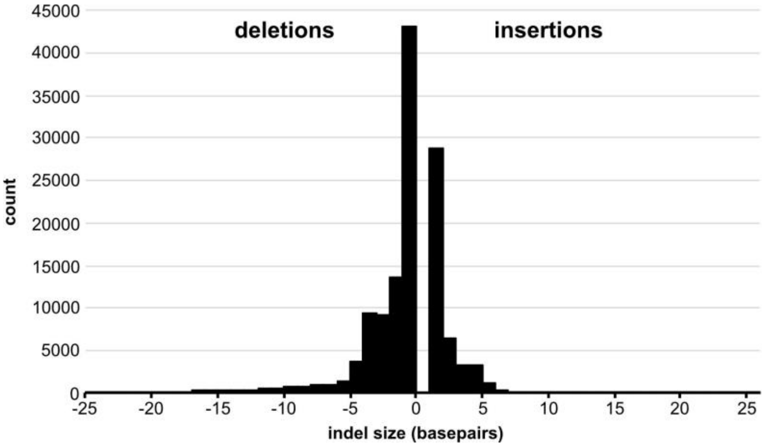

Figure S3. Size distribution and number of indels. *We identified indels using a local de-novo haplotype caller (GATK4). Only indels fixed among all five mammoth genomes and not present in any of the other elephant genomes are shown. Due to the fragmented nature of our ancient DNA reads, identifying indels larger than 25bp is challenging, thus we used a conservative cutoff of only including indels shorter than 25bp and those supported by at least five independent reads in each mammoth genome.*

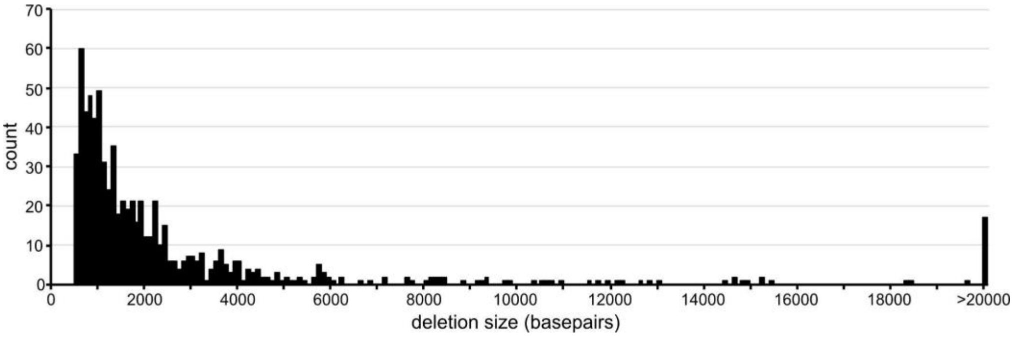

Figure S4. Size distribution and number of deletions. *Deletions of at least 500 bp in length identified using our coverage based method are shown. We included only deletions fixed among all five mammoth genomes and not present in any of the other elephant genomes. Among these, 18 deletions are above 20 kb in length, ranging up to 269 kb.*

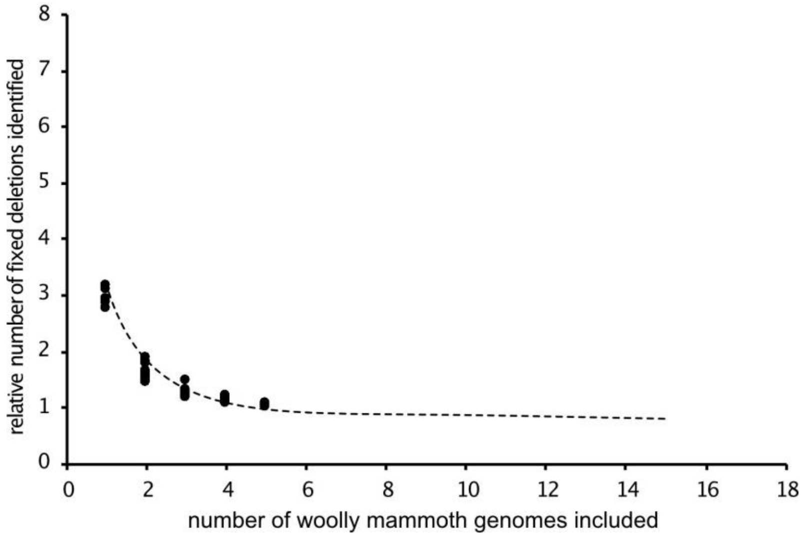

Figure S5. The effect of sample size effects on the number of fixed indels. *To estimate the fraction of deletions that are fixed in our dataset due to stochasticity we used a subsampling approach. We did so by identifying the total number of fixed, woolly mammoth specific indels when using either one, two, three, four or all five woolly mammoth genomes iterating through all possible combinations We estimated the exponential trendline using R. The y-axis shows the relative number of total indels identified as a function of the number of woolly mammoth genomes included (x-axis). Each black dot represents one subset of samples.*

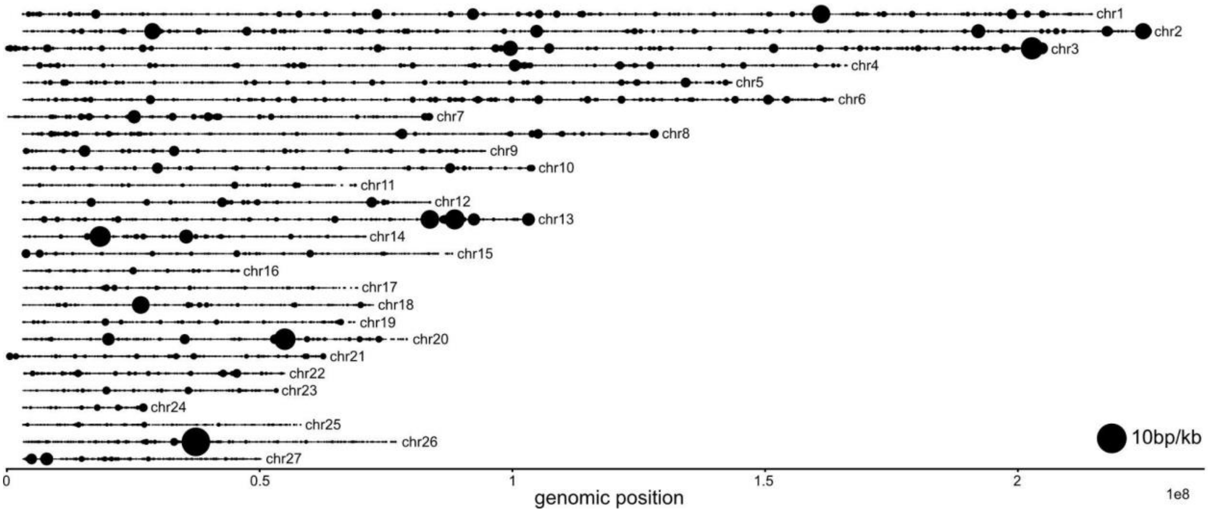

Figure S6. Deletion and indel density along the mammoth genome. *The size of the black circles represent the relative frequency of deletions and indels found in the genomic region. Deletions and indels are seen across the complete autosomal genome. Note that most of the chromosomes of the loxafr4 reference start with large stretches of unknown bases representing the telomeres, in which we could not call deletions or indels.*
